# Supplementary material for: Precision fMRI reveals that the language network exhibits adult-like left-hemispheric lateralization by 4 years of age
Source: Nat Commun. 2026 May 16;17:6505. doi: 10.1038/s41467-026-72916-5 (PMC13376885; doi:10.1038/s41467-026-72916-5)
Supplement: Supplementary file 3 — Reporting Summary [file 41467_2026_72916_MOESM3_ESM.pdf]

nature portfolio

Corresponding author(s): Ola Ozernov-Palchik

Last updated by author(s): Aug 1, 2024

Reporting Summary

Nature Portfolio wishes to improve the reproducibility of the work that we publish. This form provides structure for consistency and transparency in reporting. For further information on Nature Portfolio policies, see our Editorial Policies and the Editorial Policy Checklist.

Statistics

For all statistical analyses, confirm that the following items are present in the figure legend, table legend, main text, or Methods section.

n/a	Confirmed	
☐	☒	The exact sample size (*n*) for each experimental group/condition, given as a discrete number and unit of measurement
☐	☒	A statement on whether measurements were taken from distinct samples or whether the same sample was measured repeatedly
☐	☒	The statistical test(s) used AND whether they are one- or two-sided<br>*Only common tests should be described solely by name; describe more complex techniques in the Methods section.*
☐	☒	A description of all covariates tested
☐	☒	A description of any assumptions or corrections, such as tests of normality and adjustment for multiple comparisons
☐	☒	A full description of the statistical parameters including central tendency (e.g. means) or other basic estimates (e.g. regression coefficient) AND variation (e.g. standard deviation) or associated estimates of uncertainty (e.g. confidence intervals)
☐	☒	For null hypothesis testing, the test statistic (e.g. *F*, *t*, *r*) with confidence intervals, effect sizes, degrees of freedom and *P* value noted<br>*Give P values as exact values whenever suitable.*
☒	☐	For Bayesian analysis, information on the choice of priors and Markov chain Monte Carlo settings
☐	☒	For hierarchical and complex designs, identification of the appropriate level for tests and full reporting of outcomes
☐	☒	Estimates of effect sizes (e.g. Cohen's *d*, Pearson's *r*), indicating how they were calculated

Our web collection on statistics for biologists contains articles on many of the points above.

Software and code

Policy information about availability of computer code

Data collection	All data were collected on a 3-Tesla Siemens Prisma scanner at the Athinoula A. Martinos Imaging Center at the McGovern Institute for Brain Research at MIT.
Data analysis	fMRI analysis was performed using SPM12 (release 7487) and the CONN EvLab module (release 19b), along with custom MATLAB scripts. Statistical analyses were conducted in R v3.5.0 (R Core Team, 2013) using the lme4 package (version 1.1-22) and the lmerTest package (version 1.2-3).

For manuscripts utilizing custom algorithms or software that are central to the research but not yet described in published literature, software must be made available to editors and reviewers. We strongly encourage code deposition in a community repository (e.g. GitHub). See the Nature Portfolio guidelines for submitting code & software for further information.

Data

Policy information about availability of data

All manuscripts must include a data availability statement. This statement should provide the following information, where applicable:

- Accession codes, unique identifiers, or web links for publicly available datasets
- A description of any restrictions on data availability
- For clinical datasets or third party data, please ensure that the statement adheres to our policy

The data that support the findings of this study are available on the Open Science Framework (OSF) at the following link: https://osf.io/3mvpx/. The repository includes a visualization of individual subject maps, raw and processed data, analysis scripts, and additional materials necessary to replicate the study's results.

Access to the data is open and does not require any special permissions

Research involving human participants, their data, or biological material

Policy information about studies with human participants or human data. See also policy information about sex, gender (identity/presentation), and sexual orientation and race, ethnicity and racism.

Reporting on sex and gender	Participant sex was collected based on self-/parent-report. Gender was not collected or considered. Information for all participants and group percentages are reported in Methods. We also provide disaggregated participant-level sex in the Source Data. No sex- or gender-based analyses were performed due to a lack of a priori hypotheses.
Reporting on race, ethnicity, or other socially relevant groupings	Race and ethnicity information was collected via parent-report for the three child samples, but not the adult sample. We will add reporting of this information to Supplemental Materials section. NIH definitions and categories were used.
Population characteristics	Dataset 1: After excluding 28 child participants and 0 adult participants from group-level analyses due to excessive motion (Methods-Section5; for summary of motion outliers by group, see https://osf.io/3mvpx/), the final Dataset 1 included 206 children (ages 4-14; average = 10.69, st. dev. = 3.18; 106 females; 178 right-handed) and 91 adults (ages 19-45, average = 27.40, st. dev. = 5.5; 48 females; 84 right-handed). The pediatric data came from three projects that each focused on a different age range and did not concern the development of the language network (for details, see https://osf.io/3mvpx/?view_only=803ba79e635d4e0da1698e0a6367d7e6): early childhood (n=41; ages 4-6, average = 5.78, st. dev. = 0.69), middle childhood (n=57; ages 7-10, average = 9.00, st. dev. = 0.74), and late childhood (n=108; ages 12-14, average = 13.45, st. dev. = 0.69).<br><br>Dataset 2: After excluding 31 child participants and 0 adult participants from group-level analyses due to excessive motion (Methods-Section5; for summary of motion outliers by group, see https://osf.io/3mvpx/), the final Dataset 2 included 67 children (ages 4-16; average = 11.5, st. dev. = 1.4; 29 females) and 16 adults (ages 18-60, average = 43.8, st. dev. = 12.1; 7 females). To enable parallel analyses across the two datasets, the pediatric data in this dataset were divided into three age groups, to approximately match the division in Dataset 1: early childhood (n=6; ages 4-6, average = 5.4, st. dev. = 0.46), middle childhood (n=38; ages 7-9, average = 7.8, st. dev. = 0.65), and late childhood (n=23; ages 10-16 average = 11.5, st. dev. = 1.38).<br><br>A subset of the children and adults in Dataset 2 wore a total light-exclusion blindfold during the fMRI scan to reduce visual cortex responses to visual input during the scan, for a different study63 (children: n=14 [early = 2; middle = 7; late = 5], ages 4-16, average =8.85 , st. dev. = 2.23; adults: n=16, ages 18-59, average = 43.8, st. dev. =12.08). Blindfolded and non-blindfolded children are treated as a single group for the current study, which focuses on responses in higher-cognitive networks.
Recruitment	Child participants were recruited through outreach to local elementary schools, social media, and word of mouth. Adult participants were recruited through university listservs, flyers distributed in community centers, transit stations, and other public locations.
Ethics oversight	For all child participants, a parent or guardian provided written informed consent, and the child provided assent; adult participants provided written informed consent, in accordance with the Committee on the Use of Humans as Experimental Subjects at the Massachusetts Institute of Technology.

Note that full information on the approval of the study protocol must also be provided in the manuscript.

Field-specific reporting

Please select the one below that is the best fit for your research. If you are not sure, read the appropriate sections before making your selection.

☐ Life sciences ☒ Behavioural & social sciences ☐ Ecological, evolutionary & environmental sciences

For a reference copy of the document with all sections, see nature.com/documents/nr-reporting-summary-flat.pdf

Behavioural & social sciences study design

All studies must disclose on these points even when the disclosure is negative.

Study description	This is a quantitative, cross-sectional, experimental fMRI study.
Research sample	Dataset 1 included 206 children (ages 4–14; average = 10.69, st. dev. = 3.18; 106 females; 178 right-handed) and 91 adults (ages 19–45; average = 27.40, st. dev. = 5.5; 48 females; 84 right-handed). Data collection for the middle childhood cohort was temporarily paused for approximately one year due to disruptions related to the COVID-19 pandemic before resuming.<br><br>Dataset 2 included 67 children (ages 4-16; average = 11.5, st. dev. = 1.4; 29 females) and 16 adults (ages 18-60, average = 43.8, st. dev. = 12.1; 7 females)<br><br>Both datasets are roughly representative in terms of sex (ranging from 43% - 52% female).
Sampling strategy	Random sampling procedures were used. Sample sizes were chosen based on power analyses for each of the primary research

	questions, and constrained by financial and timing-based limitations.
Data collection	Pen + paper, fMRI data collected at McGovern Institute by a minimum of two researchers
Timing	We use five samples collected from independent studies in our institution collected between 2015-2024.
Data exclusions	Inclusion criteria: Across datasets, child and adult participants were included in the analysis based on the following criteria: 1) being a native speaker of the language used in the experiment (English for all groups except for the adult group in Dataset 1; diverse languages for the adult group in Dataset 1), 2) having no diagnosis of neurological disorder, and 3) having normal or corrected-to-normal vision. For Dataset 1, it was additionally ensured that child participants were born full-term (>37 weeks), had no history of brain injury and hospitalizations, and were not using psychotropic medications.
Non-participation	Response rate information is not consistently available across all archival datasets included in this study.
Randomization	There was no group allocation

Reporting for specific materials, systems and methods

We require information from authors about some types of materials, experimental systems and methods used in many studies. Here, indicate whether each material, system or method listed is relevant to your study. If you are not sure if a list item applies to your research, read the appropriate section before selecting a response.

Materials & experimental systems

n/a	Involved in the study	
☒	☐	Antibodies
☒	☐	Eukaryotic cell lines
☒	☐	Palaeontology and archaeology
☒	☐	Animals and other organisms
☒	☐	Clinical data
☒	☐	Dual use research of concern
☒	☐	Plants

Methods

n/a	Involved in the study	
☒	☐	ChIP-seq
☒	☐	Flow cytometry
☐	☒	MRI-based neuroimaging

Plants

Seed stocks	*Report on the source of all seed stocks or other plant material used. If applicable, state the seed stock centre and catalogue number. If plant specimens were collected from the field, describe the collection location, date and sampling procedures.*
Novel plant genotypes	*Describe the methods by which all novel plant genotypes were produced. This includes those generated by transgenic approaches, gene editing, chemical/radiation-based mutagenesis and hybridization. For transgenic lines, describe the transformation method, the number of independent lines analyzed and the generation upon which experiments were performed. For gene-edited lines, describe the editor used, the endogenous sequence targeted for editing, the targeting guide RNA sequence (if applicable) and how the editor was applied.*
Authentication	*Describe any authentication procedures for each seed stock used or novel genotype generated. Describe any experiments used to assess the effect of a mutation and, where applicable, how potential secondary effects (e.g. second site T-DNA insertions, mosiacism, off-target gene editing) were examined.*

Magnetic resonance imaging

Experimental design

Design type	task and resting state

Design specifications

Each participant completed 6-12 blocks per condition across 1-4 scanning runs. The early childhood group in Dataset 1 had a single run, manually split into two equal-sized runs for analyses. Resting state scans were performed by most participants, with durations varying by age group: 5 min 15 s for early childhood, 6 min 22 s for middle childhood, 5 min 46 s for late childhood, and 5 min for adults. Participants were excluded if more than 40% of volumes were outliers. For the language localizer task, four variants were used with different conditions and block designs:

Variant 1: Used for early childhood group (Dataset 1) - 6 experimental events per condition and 18 fixation periods, total run duration 360 s.
Variant 2: Used for middle childhood and adult groups (Dataset 1) - two versions: 6 experimental events per condition and 4 fixation periods (total run 264 s) or 4 experimental events per condition and 3 fixation periods (total run 252 s).
Variant 3: Used for late childhood group (Dataset 1) - 8 experimental events per condition and 5 fixation periods, total run duration 358 s.
Variant 4: Used for all participants (Dataset 2) - 2 experimental events per condition and 3 rest periods, total run duration 6.6 min.
The materials and specific details for each variant are available on OSF: https://osf.io/3mvpx/.

Behavioral performance measures

N/A

Acquisition

Imaging type(s)

function + structural

Field strength

3T

Sequence & imaging parameters

Dataset 1:

Early Childhood:
Structural Imaging: T1-weighted multi-echo MPRAGE, 176 sagittal slices, TR = 2,530 ms, TE = 1.64 ms/3.5 ms/5.36 ms/7.22 ms, TI = 1,400 ms, flip angle = 7°, resolution = 1.00 mm isotropic.
Functional Imaging: EPI T2*-weighted, 41 transverse slices (2 mm thick), TR = 2,500 ms, TE = 30 ms, flip angle = 90°, in-plane resolution = 3 mm × 3 mm.
Resting State: EPI T2*-weighted, 41 sagittal slices (3 mm thick), TR = 520 ms, TE = 3.41 ms/5.97 ms, flip angle = 55°, in-plane resolution = 3 mm × 3 mm.
Middle Childhood:
Structural Imaging: T1-weighted multi-echo MPRAGE, 176 sagittal slices, TR = 2,530 ms, TE = 1.69 ms/3.55 ms/5.41 ms/7.27 ms, TI = 1,400 ms, flip angle = 7°, resolution = 1.00 mm isotropic.
Functional Imaging: EPI T2*-weighted, 32 near-axial slices (4 mm thick), TR = 2,000 ms, TE = 30 ms, flip angle = 90°, in-plane resolution = 2.1 mm × 2.1 mm.
Resting State: EPI T2*-weighted, 40 sagittal slices (3 mm thick), TR = 2,500 ms, TE = 30 ms, flip angle = 90°, in-plane resolution = 3 mm × 3 mm.
Late Childhood:
Structural Imaging: T1-weighted multi-echo MPRAGE, 320 sagittal slices, TR = 4,000 ms, TE = 1.06 ms, flip angle = 2°, resolution = 2.00 mm isotropic.
Functional Imaging: EPI T2*-weighted, 72 near-axial slices (4 mm thick), TR = 1,000 ms, TE = 37.2 ms, flip angle = 63°, in-plane resolution = 2 mm × 2 mm.
Resting State: EPI T2*-weighted, 72 axial slices (2 mm thick), TR = 800 ms, TE = 37 ms, flip angle = 52°, in-plane resolution = 2 mm × 2 mm.
Adults:
Structural Imaging: T1-weighted multi-echo MPRAGE, 179 sagittal slices, TR = 2,530 ms, TE = 3.48 ms, resolution = 1 mm isotropic.
Functional Imaging: EPI T2*-weighted, 31 near-axial slices (4 mm thick), TR = 2,000 ms, TE = 30 ms, flip angle = 90°, in-plane resolution = 2.1 mm × 2.1 mm.
Dataset 2:

Structural Imaging: T1-weighted, 176 interleaved sagittal slices, TR = 2,530 ms, TE = 1.64/3.5 ms/5.36 ms/7.22 ms, resolution = 1 mm isotropic.
Functional Imaging: EPI T2*-weighted, 32 near-axial slices (3 mm thick), TR = 2,000 ms, TE = 30 ms, flip angle = 90°, in-plane resolution = 3 mm × 3 mm.

Area of acquisition

whole brain

Diffusion MRI ☐ Used ☒ Not used

Preprocessing

Preprocessing software

fMRI data were preprocessed and analyzed using SPM12 (release 7487), CONN EvLab module (release 19b) and custom MATLAB scripts

Normalization

Functional and structural data were independently normalized into a common space (the Montreal Neurological Institute (MNI) template, IXI549Space) using the SPM12 unified segmentation and normalization procedure with a reference functional image computed as the mean functional image after realignment across all time points, omitting outlier scans. The output data were resampled to a common bounding box between MNI-space coordinates (−90, −126, and −72) and (90, 90,

	and 108), using 2 mm isotropic voxels and fourth-order spline interpolation for the functional data and 1 mm isotropic voxels and tri-linear interpolation for the structural data.
Normalization template	see above
Noise and artifact removal	Participants were excluded from the language localizer task analysis if more than 40% of the acquired volumes (across both runs) were identified as outlier volumes during preprocessing. BOLD signal indicators using default thresholds in the CONN pre-processing pipeline (5 st. dev. or more above the mean in global BOLD signal change or framewise displacement values above 0.9 mm).
Volume censoring	see above

Statistical modeling & inference

Model type and settings	For the critical and control conditions of the language localizer task, effects were estimated using a general linear model (GLM) in which each experimental condition was modeled with a boxcar function convolved with the canonical hemodynamic response function (HRF) (fixation was modeled implicitly). Temporal autocorrelations in the BOLD signal timeseries were accounted for by a combination of high-pass filtering with a 128 s cutoff and whitening using an AR (0.2) model (first-order autoregressive model linearized around the coefficient a = 0.2) to approximate the observed covariance of the functional data in the context of restricted maximum likelihood (ReML) estimation. In addition to main condition effects, other model parameters in the GLM design included first-order temporal derivatives for each condition (for modeling spatial variability in the HRF delays) as well as nuisance regressors to control for the effect on the BOLD signal of slow linear drifts, subject-motion parameters, and outlier scans.
Effect(s) tested	Language>Control

Specify type of analysis: ☐ Whole brain ☒ ROI-based ☐ Both

Anatomical location(s)	For each participant, functional regions of interest (fROIs) were defined using the Group-constrained Subject-Specific (GcSS) approach31. For the language network in the left hemisphere (LH), we used five parcels derived from a group-level representation of the language localizer data in 220 adult participants (independent of the adult sample in the current study) and used in much past work (e.g.,8,50,70,98,102,105,116,117, inter alia). These parcels include three regions in the left frontal cortex (two in the inferior frontal gyrus (LIFG and LIFGorb) and one in the middle frontal gyrus (LMFG)) and two regions in the left temporal cortex (LAntTemp and LPostTemp). Individual fROIs were defined by selecting —within each parcel—the 10% of most localizer-responsive voxels based on the t-values for the Language>Control contrast (see8 for evidence that fROIs defined in this way are similar to fROIs based on a fixed statistical significance threshold). We additionally defined a set of language-responsive areas in the right hemisphere (RH). Following past work (e.g.,39), we projected the LH parcels onto the right hemisphere and selected the 10% of most localizer-responsive voxels, as in the LH. (We chose to use parcels derived from adults in order to be able to directly compare critical neural measures between children and adults, but see SI-6 for evidence that parcels derived from the pediatric data directly are similar.)

Statistic type for inference (See Eklund et al. 2016)	voxel-wise
Correction	We did not conduct corrections at the voxel or cluster level. We evaluated whether p-values for our main effects survived Bonferroni correction.

Models & analysis

n/a	Involved in the study
☐	☒ Functional and/or effective connectivity
☒	☐ Graph analysis
☒	☐ Multivariate modeling or predictive analysis

Functional and/or effective connectivity	Pearson's moment correlation
