# Supplementary material for: Precision fMRI reveals that the language network exhibits adult-like left-hemispheric lateralization by 4 years of age
Source: Nat Commun. 2026 May 16;17:6505. doi: 10.1038/s41467-026-72916-5 (PMC13376885; doi:10.1038/s41467-026-72916-5)
Supplement: Supplementary file 2 — Peer Review File [file 41467_2026_72916_MOESM2_ESM.pdf]

nature portfolio

Peer Review File

Precision fMRI reveals that the language network exhibits adult-like left-hemispheric lateralization by 4 years of age

Corresponding Author: Dr Ola Ozernov-Palchik

This file contains all reviewer reports in order by version, followed by all author rebuttals in order by version.

Version 0:

Reviewer comments:

Reviewer #1

(Remarks to the Author)
Overview
This paper addresses a simple but important question: does cerebral lateralisation for language develop in childhood, or is it already present to same extent as in adults in young children. The authors exploit two existing fMRI datasets to address this question. The literature on development of cerebral lateralisation has been rather inconsistent. It is pretty well established that the consequences of focal lesions of the left hemisphere for language are far less severe for children than for adults, but that need not mean that early language is more bilateral in children. From analysing two independent datasets, the authors argue that lateralisation is comparable to that in adults from around 4 years of age.

Evaluation
The paper is well-written with a clear rationale. The use of individualised activation maps makes good sense in the context of cerebral lateralisation, where there can be substantial individual variation. I greatly appreciate the authors making data and scripts available, though, as noted below, I could not find some key data so could not replicate the analysis. This paper could make an important contribution to the literature, helping resolve a question that has been controversial for decades, but I think there are issues with measurement and analytic approach that need fixing. It's a complex paper and I am focussing just on the main points that seem important. I have not looked at the Supplementary Material, given that I think there are big issues in the main paper that need fixing.

1. I did not find the case made by the authors entirely convincing. There were two principal issues about measurement that I think undermine the conclusions. First, the primary measure used to make the case for adult-levels of language lateralisation in children was flawed. A laterality index was computed using t-values for L and R hemisphere language activation in the formula (L-R)/(L+R). But that formula was derived for situations where in effect the resulting value is a proportion - it is a difference score that is proportional to the summed values of the measures. As the authors discovered, it cannot be applied sensibly to measures where there are negative values, such as t-values, because it can then yield LIs outside the limits of -1/+1. Their method of dealing with this was to make all values positive by adding a constant to the t-values, but this is arbitrary and the resulting LI will be very dependent on the constant. This is unlikely to yield a reproducible LI, insofar as the constant depends on the range of values observed, and this will be different, e.g. across different tasks or runs.

There are other approaches to quantifying cerebral lateralisation that do not run into this problem (see, e.g., 10.1162/nol_a_00136), but as I argue below, I'm not sure they are needed here.

2. Second, the handling of the age variable raises other issues. One option is to treat this as a category: perhaps a reasonable decision given that the age bands do form clumps and also differed somewhat on methodological grounds. If you do that, though, the categories should be recognised as being ordered, as there is an expectation that lateralisation will become greater with each age band: the data seem overanalysed if you compare each age band with all the rest. Another approach is to treat age as continuous; that seems preferable, but then I would recommend that the researchers move away from looking for a linear association. Most developmental processes show rapid development in early childhood and then level off in adulthood. You would not expect the same amount of change between 24 years and 34 years as between 4 years and 14 years. If the true functional relationship is logarithmic, and you fit a linear model, then the strength of association will be reduced - in effect the flat function in the adult range will depress the correlation.

I think both these issues can be fixed. I don't think you need a laterality index. The main null hypothesis is that the difference between left and right hemisphere activations does not change with age. This is testable in the kind of general linear model adopted elsewhere in the paper; you could use the t-value for the Language>Control contrast as the dependent variable and look at independent variable predictors of age, hemisphere and the interaction between them. This gives you in a single analysis a test of whether language is lateralised, and whether lateralisation changes with age. This would simplify the paper and reduce the number of analyses. It could be visualised using L hemisphere on the X axis, R hemisphere on Y axis and age coded symbolically - or as a facet.

3. A more difficult issue concerns statistical power. In effect, the main claim is for a lack of difference between adults and children, in terms of lateralisation. But if one focuses just on the youngest group, who arguably are best placed to show a difference, the sample size seems quite small, especially in sample 2. If we take the average sample size for youngest vs adult, then in R we can compute how well the study is powered for just a simple t-test with
power.t.test(n=66,sig.level=.05,power=.8,type="two.sample",alternative="one.sided") for sample 1, and
power.t.test(n=11,sig.level=.05,power=.8,type="two.sample",alternative="one.sided").
For sample 1, you have 80% power to detect a difference in means of d = .43; for sample 2 it is d = 1.25.
But even this is an overestimate: power to detect interactions is always lower than for differences in means - in practice, I think you'd need to do a power analysis through simulation to get a sense of the power to detect a true interaction (see, e.g., https://humburg.github.io/Power-Analysis/simr_power_analysis.html).
I realise that power issues and effect sizes tend not to be discussed in the imaging literature, but, as clearly outlined in the introduction, we have a field where there are discrepant findings; the authors make a reasonable case that this could be due to methodological differences such as measurement issues and tasks, but we do need also to consider statistical power, as when this is low, we can miss real but small effects. I don't think this precludes publication - we need to think of research on this question as a continuous process, if data are open, then the analysis here could be incorporated in a meta-analysis with future studies.

More specific points

4. At times, the theory behind the research seemed oversimplified. For instance, in the discussion, it is stated "The hypothesis whereby the language system starts out bilateral during early development makes a clear prediction that the LH and RH language areas should respond similarly to language in children, in contrast to adults". It is true that in his original work, Lenneberg saw puberty as a key point where plasticity reduced, though he noted that already at 4 years there is less plasticity than at 2 years (for a brief account: Lenneberg, E. H. (1969). On Explaining Language. Science, 164(3880), 635–643. https://doi.org/10.1126/science.164.3880.635). So I don't think he'd have predicted equipotentiality even in the youngest child groups studied here, rather a gradual progression towards the adult state. The authors go on to say of their findings "This evidence rules out the possibility that the language system is supported by a fully or more bilateral network in early childhood" (my emphasis). It may rule out a "fully bilateral network" but the problem is with the claim that it rules out a "more bilateral network" - that's much harder to demonstrate. Overall, this is far too strong a claim, especially as it rests heavily on use of a questionable laterality index and the power to detect an interaction between age and hemisphere is not strong.

5. Re open data and scripts. The availability of data and scripts was welcome, but some things were omitted, which means the work is not fully reproducible. Are the 'custom Matlab scripts' mentioned on p 24 available? I downloaded 'code_data_to_share' from OSF and was pleased to find data and well-commented scripts, but when I tried to run 'Alice_final_analyses_w_figures_050624.Rmd' I fell at the first hurdle, as I could not find the first file that is downloaded with this command:
demo_data <- read.csv('./all_demographics_and_exclusion050923.csv')

Minor issues
On P 4 this section was confusing: "To foreshadow the results, across both datasets, we found that left-hemisphere lateralization based on (1) response magnitude and (2) volume of activation was already adult-like by four years of age (see SI-10 for generalization to a third, previously published dataset25). In contrast, other aspects of the brain organization for language show a clear developmental trajectory: both the magnitude of response to language...." I assume that what is intended here is to draw a distinction between magnitude of response to language in the left hemisphere, and lateralisation of that response. Some rewording should make it clearer.

References: the formatting of titles varies between sentence case and title case and all capitals. DOIs would be nice (assuming this is permitted by the journal).

I guess it may be a requirement of the journal, but I would like to just record my deep dislike for papers that put Methods at the end. I need to read the Methods first to understand the results, and I end up flipping to and fro to find what I want. Also, there is a description of the localiser task in the Results that should be in Methods, but was presumably put in Results to make the paper intelligible given the sequence of sections: evidence enough that we need to know what was done before we can make sense of what was found. (While I am at it, I also hate numbered references, as I have to keep flipping to back of the paper to find what is being referred to, but that's definitely a gripe for the publisher and not for the authors).

Dorothy Bishop
1 August 2024

Reviewer #2

(Remarks to the Author)
This thorough and well written study uses a large combined data set from functional MRI in children through adults (i.e. a large cross-sectional sample) to challenge the prior literature suggesting that left-lateralization of the neural mechanisms for language processing is minimal in children and increases by adulthood. The results presented suggest that left lateralization is already present at preschool age and continues in a similar pattern across development. The study's strengths include the large sample size, a well justified preprocessing approach for the fMRI data, and detailed descriptions of the language tasks and statistical methods, including a comparison with an existing study in the literature included in the supplementary materials. This study overall makes an important contribution to the field, however, I had some concerns with the the authors initial framing of concepts related to language processing and lateralization, and I also had questions about the specific method for region of interest selection in the statistical analysis.

Specific comments are as follows:

Introduction:
- While I understand space in the manuscript is limited, I feel the others are underrepresenting the complexity of the multiple levels of representation involved in language processing. While some previous studies refer to the neural system being studied in this manuscript as "the language system", the task versus control comparisons that the current study is built on engage a number of processes including recognition of speech sounds, word recognition, processing of syntax, and semantic interpretation. This should be clearly stated.
- On a related note, I find it odd that the authors talk about language processing being conflated with speech perception because speech perception is fundamental to the comprehension of spoken language, and the control conditions compared to language stimuli in this study do not entirely subtract out speech perception (see my comments on the methods below). This should be clarified in the introduction.
- In addition, I think the others are overstating previous claims that left-lateralization of some aspects of language processing could not be present in preschool-aged children. Assuming that the hemispheres are equipotential at birth does not rule out the possibility that lateralization could begin very early in life and increase in degree across development. The brain injury/stroke literature in this area I think clearly recognizes that while there is great potential for plasticity and recovery using increased engagement of right hemisphere mechanisms for language, this is not necessarily equal to typical development in the left hemisphere. So the authors should be clear that a fully bilateral language system at preschool age is not necessarily a prevailing idea in the existing literature.

Results:
The results are overall very clearly presented and the different metrics for assessing lateralization are a strength of the manuscript. My concerns lie primarily with the methods, which I will detail below.

Discussion:
- As in the introduction, I feel that the authors are overstating the state of the existing literature regarding trends of language lateralization from childhood to adulthood. Existing studies in this area recognize that there is bilateral engagement of relevant brain regions in both children and adults, while still suggesting an increase in *degree* of left lateralization across development (not confirmed in the current study).
- The authors make an important point that bilateral activation observed in many studies is likely to be associated with increased task demands. However, I am unsure that it is possible with any task to completely separate increased task demands from increased reliance on language processing, and it could be helpful for the authors to elucidate their approach to doing this (some references are given but it would be helpful to have more information in the discussion if space allows.)
- Related to this, because there were not consistent measures of comprehension associated with the different language tasks, it is difficult to assess to what extent participants were attending to the stimuli, which is likely to affect activation patterns.
- The authors also mention the specialization of the right hemisphere for non-linguistic information however they should also consider that the right hemisphere has been shown to process other components of spoken language, such as voice recognition and some types of prosody. (The work of Hickok and Poeppel is highly relevant to this discussion and should be cited).
- In addition, the discussion of continued language development across the age range included in the study is somewhat sparse. There are specific language skills that continue to develop at different ages. While speech perception and basic syntactic skills are well in place by preschool age, higher level language skills such as inferencing continue to develop, as well as dramatic increases in vocabulary.

Methods:
- While I think it is reasonable to combine the different language stimuli and control conditions as the authors have done, it is important for them to clarify that the contrasts are *not* the same across the datasets. Importantly, backward speech contains limited recognizable phonemic information. Degraded speech, as described in the methods, is also unlikely to include recognizable phonemic information this means that when comparing these conditions to normal speech, recognition of speech sounds is a part of the activation in that comparison. In contrast, when using a foreign language as the control condition it is likely that there is overlap in phonemic information in the native language in foreign language.
- The task in dataset two included a probe condition, but performance data is not included and it is unclear whether this varied with age.

- While the analysis approach is generally well motivated, I am concerned about the effect of choosing the top 10% of activated voxels within each parcel as individually-defined functional ROIs. This could be clarified with some examples of how this would look on an individual level and some quantitative data on numbers of voxels involved (were they always contiguous within the parcel?). Are the sample activation maps included in Figure 2 thresholded based on this top 10% criteria? It seems like excluding a large number of voxels in each parcel would have a dramatic effect on lateralization calculations, and restricting the analysis to the most active voxels in the parcels in both hemispheres might bias the results. The overlap maps shown in Figure SI-6 do suggest that areas of activation in children largely overlapped with those of adults, but it is unclear how spatially consistent this was at the individual level, and how this would affect lateralization calculations. Much clarification is needed on this aspect of the methods.

- It is briefly noted that not all participants were native speakers of English and some participants were tested in other languages. In some ways this is a strength of the study because the results are not limited to English, however this language diversity was included only for adult participants and not for children, so it represents a confound when looking at potential changes across age. In addition, the degree of bilingualism of participants is not described.

Reviewer #3

(Remarks to the Author)
Overview:
This paper reports on an investigation of language localization in predominantly English-speaking individuals aged between 4 and 60 years. Localization was based on three passive (i.e., listening) and one more active (comprehension: Yes-No decision to consistency of story segments) tasks. Overall results suggest similar bias across multiple age groups.
Dear Dr Ozernof-Palchik and colleagues,
Thank you for the complex and thorough work. It's great to see people approaching this question from multiple angles. The work appears to be carefully conducted, and the paper is clearly written, well presented, and will be a great contribution to the field. I have a few general comments and a series of specific comments that may have a bearing on the presentation of the work. I hope these are helpful.
Best wishes,
General comments:
1. Language network vs. listening/comprehension
Given the language listening and comprehension tasks used, it would be worth considering whether the general claims about the language network should be tempered. The justification of the use of these tasks over productive tasks is sound, but given the breadth of the language system (Poeppel, 2003; Poeppel et al., 2012; Poeppel & Hickok, 2004), being specific about the claims feels important.
2. The broader language network
In addition to the above comment, having multiple tasks to interrogate for the language network would be a helpful consideration for future work (Parker et al., 2022). There may be some covert production tasks that could be used in future research (Bookheimer et al., 1998; Wilke et al., 2005, 2010). Consider including reference to these in the current discussion.
3. LI calculations
It would be useful to know whether the methods developed by Wilke and Lidzba (2007) were considered for the LI calculation. It feels like a robust technique that would be appropriate for this work.
4. Lateralisation development and neuropsychology
The introduction states that bilateral language activation during early childhood has been proposed because early left-hemispheric brain damage can leave language relatively unimpaired. However, all but one of the studies cited (references 13-18) investigate children with damage acquired before or within one year of birth. Since this studies' sample are 4+ years old, its findings don't seem to contradict the possible bilateral language representation as much as the discussion suggests it does (line 434). The samples' being too old is mentioned briefly in the discussion, but I would suggest either including age of early left-hemisphere damage being generally below four as an explanation for the discrepancy, or highlighting examples where the damage occurred closer to the age of 4.
5. Methods for estimating lateralisation
Three methods of estimating lateralisation based on the fMRI data are included, but it would be good to see some rationale/explanation for why all three methods were used, and maybe some assessment of the merits of each for assessing lateralisation. Something to consider is that voxel counting might lead to an overestimation of the degree of lateralisation (Bradshaw et al., 2017; Jansen et al., 2006).
Specific comments:
6. Page 21, line 596: "After excluding 31 child participants and 0 adult participants" > the exclusion of 0 adults is mentioned here for Dataset 2. For consistency, it would be best to mention with for Dataset 1 as well (line 586)
7. Page 27, lines 861-862: "For magnitude values, there were two subjects with negative values (one in DS1 and one in DS2)." > This sounds like only 2 subjects were right lateralized for language processing. We would expect more than this in a random sample. Please check to ensure that this is accurate. Alternatively, please clarify in text if I have misinterpreted this value.
8. Page 27, lines 862-864: "To allow for LI calculation, we transformed the mean values for each hemisphere by adding the absolute value of the lowest negative number across all fROIs to all values separately for DS1 and DS2." > Can you clarify in text why this transformation was necessary?
9. Page 12, Figure 3 > It would be helpful to match the y-axes for the top two panels, magnitude for Datasets 1 and 2.
10. Page 12, line 296: "we used the magnitude of the top 10% of tvalues from the Language>Control" > hyphen missing in 't-

values' here: just flagging, could be a personal preference or journal requirement. Please check.
11. Page 22, line 556: "developmental brain disorders104,105 (see106 for a meta-analysis)" > given that these papers exclusively refer to autism spectrum disorder, the sentence might as well specify this group.
12. Page 3, lines 91-92: The sample is described as a 'pediatric cohort' throughout the text, but both datasets include child and adult participants, so that is not an appropriate label.
13. Page 21, 578-584: It is also not clear if the participants were recruited from a patient population, which 'pediatric' seems to imply. There should be more details about how each dataset was recruited.

References

Bookheimer, S. Y., Zeffiro, T. A., Blaxton, T. A., Gaillard, W. D., Malow, B., & Theodore, W. H. (1998). Regional cerebral blood flow during auditory responsive naming: Evidence for cross-modality neural activation. Neuroreport, 9(10), 2409–2413.
Bradshaw, A. R., Bishop, D. V. M., & Woodhead, Z. V. J. (2017). Methodological considerations in assessment of language lateralisation with fMRI: A systematic review. PeerJ, 5, e3557. https://doi.org/10.7717/peerj.3557
Jansen, A., Menke, R., Sommer, J., Förster, A. F., Bruchmann, S., Hempleman, J., Weber, B., & Knecht, S. (2006). The assessment of hemispheric lateralization in functional MRI—Robustness and reproducibility. NeuroImage, 33(1), 204–217. https://doi.org/10.1016/j.neuroimage.2006.06.019
Parker, A. J., Woodhead, Z. V. J., Carey, D. P., Groen, M. A., Gutierrez-Sigut, E., Hodgson, J., Hudson, J., Karlsson, E. M., MacSweeney, M., Payne, H., Simpson, N., Thompson, P. A., Watkins, K. E., Egan, C., Grant, J. H., Harte, S., Hudson, B. T., Sablik, M., Badcock, N. A., & Bishop, D. V. M. (2022). Inconsistent language lateralisation—Testing the dissociable language laterality hypothesis using behaviour and lateralised cerebral blood flow. Cortex. https://doi.org/10.1016/j.cortex.2022.05.013
Poeppel, D. (2003). The analysis of speech in different temporal integration windows: Cerebral lateralization as 'asymmetric sampling in time'. Speech Communication, 41(1), 245–255. https://doi.org/10.1016/S0167-6393(02)00107-3
Poeppel, D., Emmorey, K., Hickok, G., & Pylkkänen, L. (2012). Towards a New Neurobiology of Language. The Journal of Neuroscience, 32(41), 14125–14131. https://doi.org/10.1523/JNEUROSCI.3244-12.2012
Poeppel, D., & Hickok, G. (2004). Towards a new functional anatomy of language. Cognition, 92(1–2), 1–12. https://doi.org/10.1016/j.cognition.2003.11.001
Wilke, M., & Lidzba, K. (2007). LI-tool: A new toolbox to assess lateralization in functional MR-data. Journal of Neuroscience Methods, 163(1), 128–136. https://doi.org/10.1016/j.jneumeth.2007.01.026
Wilke, M., Lidzba, K., Staudt, M., Buchenau, K., Grodd, W., & Krägeloh-mann, I. (2005). Comprehensive language mapping in children, using functional magnetic resonance imaging: What's missing counts. Neuroreport, 16(9), 915–919.
Wilke, M., Pieper, T., Lindner, K., Dushe, T., Holthausen, H., & Krägeloh-Mann, I. (2010). Why one task is not enough: Functional MRI for atypical language organization in two children. European Journal of Paediatric Neurology, 14(6), 474–478. https://doi.org/10.1016/j.ejpn.2010.05.002

Version 1:

Reviewer comments:

Reviewer #1

(Remarks to the Author)
The authors have been highly responsive to suggestions for revision and incorporated additional analyses in their revision. I have also looked at the deposited code and data which allow one to reconstruct the analyses. That is greatly appreciated. Just to add, re statistical power - I was not recommending a post hoc power analysis in my original report, but rather using simulation to obtain power estimates. Apologies if that was unclear. Although that was not done but I am happy to go ahead and recommend publication: this study clearly advances our understanding of this topic.

(Remarks on code availability)
I have not worked through all the code but have satisfied myself that the results are replicable using the deposited data and code.

Reviewer #2

(Remarks to the Author)
Overall, the authors have been highly responsive to the reviews and I am enthusiastic about seeing this work move forward for publication. I had one minor point remaining:

- The revision clarified my understanding about selection of the top 10% of voxels in each ROI, however, I still feel the explanation in the methods section is confusing. On page 23 of the main document, the authors state "Individual fROIs were defined by selecting—within each parcel—the 10% of most localizer-responsive voxels based on the t-values for the Language>Control contrast". To me this implied that only *10% of the voxels in each parcel* were selected, which led to my original concern about this method. In the supplementary information, the authors explain that they "selected the top 10% of voxels across the brain for the Language > Control contrast" and then divided up the selected regions according to each parcel. I think the explanation in the supplementary information is clearer and should be carried over to the main manuscript.

(Remarks on code availability)
Verified that code was available with README, did not run it myself

Open Access This Peer Review File is licensed under a Creative Commons Attribution 4.0 International License, which permits use, sharing, adaptation, distribution and reproduction in any medium or format, as long as you give appropriate credit to the original author(s) and the source, provide a link to the Creative Commons license, and indicate if changes were made.
In cases where reviewers are anonymous, credit should be given to 'Anonymous Referee' and the source.
The images or other third party material in this Peer Review File are included in the article's Creative Commons license, unless indicated otherwise in a credit line to the material. If material is not included in the article's Creative Commons license and your intended use is not permitted by statutory regulation or exceeds the permitted use, you will need to obtain permission directly from the copyright holder.
To view a copy of this license, visit https://creativecommons.org/licenses/by/4.0/

Reviewer #1 (Remarks to the Author):

Overview

This paper addresses a simple but important question: does cerebral lateralisation for language develop in childhood, or is it already present to same extent as in adults in young children. The authors exploit two existing fMRI datasets to address this question. The literature on development of cerebral lateralisation has been rather inconsistent. It is pretty well established that the consequences of focal lesions of the left hemisphere for language are far less severe for children than for adults, but that need not mean that early language is more bilateral in children. From analysing two independent datasets, the authors argue that lateralisation is comparable to that in adults from around 4 years of age.

Evaluation

The paper is well-written with a clear rationale. The use of individualised activation maps makes good sense in the context of cerebral lateralisation, where there can be substantial individual variation. I greatly appreciate the authors making data and scripts available, though, as noted below, I could not find some key data so could not replicate the analysis. This paper could make an important contribution to the literature, helping resolve a question that has been controversial for decades, but I think there are issues with measurement and analytic approach that need fixing. It's a complex paper and I am focussing just on the main points that seem important. I have not looked at the Supplementary Material, given that I think there are big issues in the main paper that need fixing.

Thank you for recognizing the value of this work and for very helpful comments – we address those below.

R1.1: *I did not find the case made by the authors entirely convincing. There were two principal issues about measurement that I think undermine the conclusions. First, the primary measure used to make the case for adult-levels of language lateralisation in children was flawed. A laterality index was computed using t-values for L and R hemisphere language activation in the formula (L-R)/(L+R). But that formula was derived for situations where in effect the resulting value is a proportion - it is a difference score that is proportional to the summed values of the measures. As the authors discovered, it cannot be applied sensibly to measures where there are negative values, such as t-values, because it can then yield LIs outside the limits of -1/+1. Their method of dealing with this was to make all values positive by adding a constant to the t-values, but this is arbitrary and the resulting LI will be very dependent on the constant. This is unlikely to yield a reproducible LI, insofar as the constant depends on the range of values observed, and this will be different, e.g. across different tasks or runs.*

There are other approaches to quantifying cerebral lateralisation that do not run into this problem (see, e.g., 10.1162/nol_a_00136), but as I argue below, ***I'm not sure they are needed*** *here.*

RESPONSE R1.1: Thank you for these comments, we appreciate the limitations of the LI measure and have addressed them as recommended in **R1.2** below.

***R1.2**: Second, the handling of the age variable raises other issues. One option is to treat this as a category: perhaps a reasonable decision given that the age bands do form clumps and also differed somewhat on methodological grounds. If you do that, though, the categories should be recognised as being ordered, as there is an expectation that lateralisation will become greater with each age band: the data seem overanalysed if you compare each age band with all the rest. Another approach is to treat age as continuous; that seems preferable, but then I would recommend that the researchers move away from looking for a linear association. Most developmental processes show rapid development in early childhood and then level off in adulthood. You would not expect the same amount of change between 24 years and 34 years as between 4 years and 14 years. If the true functional relationship is logarithmic, and you fit a linear model, then the strength of association will be reduced - in effect the flat function in the adult range will depress the correlation.*

I think both these issues can be fixed. I don't think you need a laterality index. The main null hypothesis is that the difference between left and right hemisphere activations does not change with age. This is testable in the kind of general linear model adopted elsewhere in the paper; you could use the t-value for the Language>Control contrast as the dependent variable and look at independent variable predictors of age, hemisphere and the interaction between them. This gives you in a single analysis a test of whether language is lateralised, and whether lateralisation changes with age. This would simplify the paper and reduce the number of analyses.

RESPONSE R1.2:

Thank you for this insightful suggestion. We agree with all the points you raise, and have now adopted your proposed solution for our main analysis of lateralization effects. In particular, we have run a linear mixed-effects model using the following formula: *Language>Control contrast ~ hemisphere* × *age,* with by-subject random intercepts and hemisphere slopes, and by-ROI random intercepts and hemisphere slopes (allowing ROI-specific hemisphere effects and cross-hemisphere correlations). In terms of how to code the age predictor in the proposed model, you have pointed out the pros and cons of categorical versus continuous coding. You have suggested that for the categorical coding of age group, you would use ordered categories, but this approach runs into the same issue as the continuous coding of age given that linearity would be assumed (unless polynomial rather than simple ordered coding is used, but polynomial coding has its own suite of complexities). As a result, for the **main analysis**, we used categorical coding for age, with the contrasts set up in a way such that each child group is compared to the adult group. This choice of contrasts corresponds most closely to the claim that we would like to make: namely, that *already in the youngest group, language processing is lateralized to the left hemisphere at least as strongly as in adults (i.e., that no difference exists between adults and youngest children in the degree of language lateralization (or between adults and other developmental groups)).* As shown in our new **Table 3** (line 265) and **Figure 3** (line 277), we do not see significant age x hemisphere interactions between any developmental group and the adult group in either dataset. To demonstrate **robustness to analytic choices**, we additionally report the results for the same model but with the age predictor coded as an ordered categorical variable (new section **SI-3F;** SI line 419), which shows similar results (no significant interactions between age and hemisphere), and a Bayesian version of this analysis, which shows substantial-to-strong evidence for the null hypothesis (new section **SI-3B;** line 226). Finally, to leverage the large total number of

participants in our study, we ran our main model on the combined dataset (new section **SI-3D;** SI line 372) and found the same results.

For comparability with many past developmental investigations of language lateralization (Vingerhoets et al., 2023), we chose to retain the LI measure based on language region volumes (which does not run into the issue of negative values that you commented on); however, to simplify the analyses and increase statistical power, we now use a single LI per participant (collapsing across ROIs). The results again show no age-related increases in lateralization. Following a recommendation from Reviewer 3 (**R3.3**), we have also added an analysis of lateralization using the LI Toolbox available for SPM (reported in **SI-3C;** SI line 348). This toolbox employs adaptive thresholding and bootstrapping techniques to reduce the impact of outliers, specific thresholds, and data sparsity on the LI calculation (Wilke & Lidzba, 2006; Wilke & Lidzba, 2007). This approach once again finds no age-related increase in language lateralization. (Brief results in the main manuscript: lines 260-264; Brief methods in the main manuscript: lines 777-779; 825-827.)

R1.3: *A more difficult issue concerns statistical power. In effect, the main claim is for a lack of difference between adults and children, in terms of lateralisation. But if one focuses just on the youngest group, who arguably are best placed to show a difference, the sample size seems quite small, especially in sample 2. If we take the average sample size for youngest vs adult, then in R we can compute how well the study is powered for just a simple t-test with power.t.test(n=66,sig.level=.05,power=.8,type="two.sample",alternative="one.sided") for sample 1, and power.t.test(n=11,sig.level=.05,power=.8,type="two.sample",alternative="one.sided"). For sample 1, you have 80% power to detect a difference in means of d = .43; for sample 2 it is d = 1.25.*

But even this is an overestimate: power to detect interactions is always lower than for differences in means - in practice, I think you'd need to do a power analysis through simulation to get a sense of the power to detect a true interaction (see, e.g., https://humburg.github.io/Power-Analysis/simr_power_analysis.html).
I realise that power issues and effect sizes tend not to be discussed in the imaging literature, but, as clearly outlined in the introduction, we have a field where there are discrepant findings; the authors make a reasonable case that this could be due to methodological differences such as measurement issues and tasks, but we do need also to consider statistical power, as when this is low, we can miss real but small effects. I don't think this precludes publication - we need to think of research on this question as a continuous process, if data are open, then the analysis here could be incorporated in a meta-analysis with future studies.

RESPONSE R1.3:

Thank you for raising this important point about the distinction between non-significant results and evidence for a null effect. We absolutely agree that absence of evidence is not evidence of absence, and we have addressed this issue in the current revision.

However, we chose an alternative approach to the post-hoc power analysis approach that you recommended. The reason is that post-hoc power analyses do not generate new insights: they simply repeat the effects of the current sample, such that whatever came out as a significant effect will have high power, and whatever came out as non-significant will have low power (for discussions of this point, see Hoenig & Heisey, 2001; Lakens, 2022; Levine & Ensom, 2001; Senn, 2002; Zhang et al., 2019; Albers & Lakens, 2018; O'Keefe, 2007). Intuitively, when we ask about power, we ask whether we can detect that a parameter is different from 0—a simple binary decision. However, to estimate power, we need precise estimates of many parameters (effect sizes, variances, and covariances). Making decisions about these estimates is far from trivial and often not justified.

Instead, we opted for an analysis of Bayes Factors, which can be used to estimate the strength of the evidence for a null hypothesis and which is generally recommended by statisticians over the post-hoc power analysis (Wagenmakers, 2007; Dienes, 2014). This analysis is now reported in **SI-3B** (SI line 226). We fit several models to evaluate different hypotheses. For example, we evaluated the hypothesis that lateralization changes monotonically across development (either increasing or decreasing with age, but not necessarily linearly). The Bayes Factors from Dataset 1 indicated that a null effect was more likely than the alternative (BF01 ranging from 1.84 to 12.67, where values >1 indicate evidence favoring the null hypothesis). The Bayes Factors from Dataset 2 were inconclusive, likely due to the smaller sample sizes in some age groups, but in line with the main analyses, all the interaction effect sizes were very small relative to other effects in the model (e.g., activation in response to language in the left hemisphere). Thus, we can rule out the possibility of a meaningful monotonic change in lateralization across development. We also evaluated the hypothesis of differences in lateralization comparing each child group to adults without imposing monotonic constraints. Neither dataset contained substantial evidence for the inclusion of age x hemisphere interaction whereby the effect of hemisphere is larger in children compared to adults. A short summary of these methods, results, and discussion are also included in the main manuscript (line 257; line 785; line 372)

Taken together, these results indicate that lateralization either remains constant across development or changes so little that it is of minimal theoretical importance.

More specific points

R1.4: *At times, the theory behind the research seemed oversimplified. For instance, in the discussion, it is stated "The hypothesis whereby the language system starts out bilateral during early development makes a clear prediction that the LH and RH language areas should respond similarly to language in children, in contrast to adults". It is true that in his original work, Lenneberg saw puberty as a key point where plasticity reduced, though he noted that already at 4 years there is less plasticity than at 2 years (for a brief account: Lenneberg, E. H. (1969). On Explaining Language. Science, 164(3880), 635–643. https://doi.org/10.1126/science.164.3880.635). So I don't think he'd have predicted equipotentiality even in the youngest child groups studied here, rather a gradual progression towards the adult state. The authors go on to say of their findings "This evidence rules out the possibility that the language system is supported by a fully or more bilateral network in early childhood" (my emphasis). It may rule out a "fully bilateral network" but the problem is with the*

claim that it rules out a "more bilateral network" - that's much harder to demonstrate. ***Overall, this is far too strong a claim, especially as it rests heavily on use of a questionable laterality index and the power to detect an interaction between age and hemisphere is not strong.***

RESPONSE R1.4:

We have addressed the concerns regarding the laterality index and power above, in **responses R1.2** and **R1.3**, respectively. We also agree with your reading of Lenneberg and have dampened our claims throughout the Introduction (e.g., lines 64-65; line 69) and Discussion (e.g., line 363; 424; 530). If you notice any residual places where something seems overstated, please let us know.

R1.5: *Reopen data and scripts. The availability of data and scripts was welcome, but some things were omitted, which means the work is not fully reproducible. Are the 'custom Matlab scripts' mentioned on p 24 available? I downloaded 'code_data_to_share' from OSF and was pleased to find data and well-commented scripts, but when I tried to run 'Alice_final_analyses_w_figures_050624.Rmd' I fell at the first hurdle, as I could not find the first file that is downloaded with this command:*
demo_data <- read.csv('./all_demographics_and_exclusion050923.csv')

RESPONSE R1.5:

Thank you for pointing out this issue, and we apologize for the error. We have relocated the misplaced file to the correct directory. To ensure the issue was fully resolved, we had someone outside our research team run the code independently, and we have addressed all residual issues.

Minor issues:
R1.6: *On P 4 this section was confusing: "To foreshadow the results, across both datasets, we found that left-hemisphere lateralization based on (1) response magnitude and (2) volume of activation was already adult-like by four years of age (see SI-10 for generalization to a third, previously published dataset [25]). In contrast, other aspects of the brain organization for language show a clear developmental trajectory: both the magnitude of response to language...." I assume that what is intended here is to draw a distinction between magnitude of response to language in the left hemisphere, and lateralisation of that response. Some rewording should make it clearer.*

RESPONSE R1.6:

Thank you, and yes, your interpretation is correct. We have clarified the wording in the relevant section (line 108).

R1.7: *References: the formatting of titles varies between sentence case and title case and all capitals. DOIs would be nice (assuming this is permitted by the journal).*

RESPONSE R1.7:

Thank you—we have fixed the reference formatting and have included DOIs.

***R1.8**: I guess it may be a requirement of the journal, but I would like to just record my deep dislike for papers that put Methods at the end. I need to read the Methods first to understand the results, and I end up flipping to and fro to find what I want. Also, there is a description of the localiser task in the Results that should be in Methods, but was presumably put in Results to make the paper intelligible given the sequence of sections: evidence enough that we need to know what was done before we can make sense of what was found. (While I am at it, I also hate numbered references, as I have to keep flipping to back of the paper to find what is being referred to, but that's definitely a gripe for the publisher and not for the authors).*

RESPONSE R1.8:

We hear you and also frequently complain about these issues; however, we have maintained the structure and reference format in order to adhere to the journal's requirements.

Dorothy Bishop
1 August 2024

Reviewer #2 (Remarks to the Author):

This thorough and well written study uses a large combined data set from functional MRI in children through adults (i.e. a large cross-sectional sample) to challenge the prior literature suggesting that left-lateralization of the neural mechanisms for language processing is minimal in children and increases by adulthood. The results presented suggest that left lateralization is already present at preschool age and continues in a similar pattern across development. The study's strengths include the large sample size, a well justified preprocessing approach for the fMRI data, and detailed descriptions of the language tasks and statistical methods, including a comparison with an existing study in the literature included in the supplementary materials. This study overall makes an important contribution to the field, however, I had some concerns with the the authors initial framing of concepts related to language processing and lateralization, and I also had questions about the specific method for region of interest selection in the statistical analysis.

Thank you for your positive assessment and for really helpful comments, which we address in the revision (and summarize below).

Specific comments are as follows:

Introduction:
***R2.1**: While I understand space in the manuscript is limited, I feel the authors are underrepresenting the complexity of the multiple levels of representation involved in language processing. While some previous studies refer to the neural system being studied in this manuscript as "the language system", the task versus control comparisons that the current study is built on engage a number of processes including recognition of speech sounds, word recognition, processing of syntax, and semantic interpretation. This should be clearly stated.*

RESPONSE R2.1:

Thank you for this comment—we absolutely agree that language processing is complex and requires a host of computations at different grains of language structure. We have now more explicitly acknowledged this complexity when introducing the contrast at the beginning of the Results section (lines 128-129; 132).

It is also worth noting that the processes that our localizer contrast encompasses appear to *not be spatially dissociable*. In particular, the fronto-temporal language network appears to support computations related to lexical retrieval, syntactic structure building, and semantic composition in both comprehension and production, and all of these processes are distributed across this network (the large body of evidence for this claim was recently summarized by our group in Fedorenko, Ivanova, and Regev (2024, *Nat Rev Neurosci*). Moreover, a network identified by the language localizer contrasts, like the ones we use here, can be recovered at the individual level without any contrasts: from voxel-wise patterns of functional connectivity, and the networks recovered in this purely data-driven way correspond closely to the networks identified with our localizer (e.g., see Braga et al., 2020 *J Neurophys*; Du et al., 2024 *J Neurophys* and 2025 *bioRxiv*; Shain & Fedorenko, 2025 *bioRxiv*). This is all to say that the language localizer that we use is simply a quick and efficient way to identify the relevant functional subset of the brain.

R2.2: *On a related note, I find it odd that the authors talk about language processing being conflated with speech perception because speech perception is fundamental to the comprehension of spoken language, and the control conditions compared to language stimuli in this study do not entirely subtract out speech perception (see my comments on the methods below). This should be clarified in the introduction.*

RESPONSE R2.2:

Thank you for the chance to clarify this point. Speech perception is absolutely fundamental to comprehension of spoken language. However, in the adult brain, speech perception relies on brain areas that are robustly distinct from the brain areas that support language comprehension. Speech perception appears to rely on regions in bilateral auditory cortex, which are highly selective for speech sounds relative to other kinds of sounds (e.g., Norman-Haignere et al., 2015, *Neuron*) and have a characteristic temporal integration window (e.g., Overath et al., 2015, *Nat Neuro*), but critically, these regions are not sensitive to *whether the signal is interpretable*: for example, they respond similarly strongly to speech in one's own language and speech in an unfamiliar foreign language or to carefully acoustically-matched controls. In contrast, language comprehension—across listening and reading (and signing in sign language users, for that matter)—relies on the left-lateralized network (what we call "the language network"). We discuss the evidence for this separation between the speech processing brain areas and the language comprehension areas in the review mentioned in the previous response (Fedorenko, Ivanova, & Regev, 2024, *NRN*, see section "*The language network and language-relevant perceptual and premotor areas*"). Most relevantly for the purposes of the current paper, speech perception mechanisms appear to be bilateral (if they show a hemispheric bias, it is much smaller than the bias for the language-processing mechanisms; e.g., Norman-Haignere et al., 2015

Neuron); as a result, contrasts that do not subtract the speech-perceptual components may result in more bilateral-appearing activations. We have clarified this dissociation, including some critical references in the Introduction (lines 81-86).

R2.3: *In addition, I think the authors are overstating previous claims that left-lateralization of some aspects of language processing could not be present in preschool-aged children. Assuming that the hemispheres are equipotential at birth does not rule out the possibility that lateralization could begin very early in life and increase in degree across development. The brain injury/stroke literature in this area I think clearly recognizes that while there is great potential for plasticity and recovery using increased engagement of right hemisphere mechanisms for language, this is not necessarily equal to typical development in the left hemisphere. So the authors should be clear that a fully bilateral language system at preschool age is not necessarily a prevailing idea in the existing literature.*

RESPONSE R2.3:

Yes, thank you. This comment echoes Reviewer 1, too. We have now clarified this point in both the Introduction (e.g., lines 64-65; line 69) and Discussion (e.g., line 363; 424; 530).

Results:
The results are overall very clearly presented and the different metrics for assessing lateralization are a strength of the manuscript. My concerns lie primarily with the methods, which I will detail below.

Discussion:
R2.4: *As in the introduction, I feel that the authors are overstating the state of the existing literature regarding trends of language lateralization from childhood to adulthood. Existing studies in this area recognize that there is bilateral engagement of relevant brain regions in both children and adults, while still suggesting an increase in *degree* of left lateralization across development (not confirmed in the current study).*

RESPONSE R2.4:

Thank you. We have now clarified this point in both the Introduction (e.g., lines 64-65; line 69) and Discussion (e.g., line 363; 424; 530).

R2.5: *The authors make an important point that bilateral activation observed in many studies is likely to be associated with increased task demands. However, I am unsure that it is possible with any task to completely separate increased task demands from increased reliance on language processing, and it could be helpful for the authors to elucidate their approach to doing this (some references are given but it would be helpful to have more information in the discussion if space allows.)*

RESPONSE R2.5:

Thank you for the invitation to clarify this point. We have now briefly expanded on this issue in the Discussion (lines 408-414).

Briefly: passive comprehension appears to selectively engage the language network, eliciting no response (above the fixation/silence baseline) in the brain network that is robustly sensitive to task demands (i.e., the Multiple Demand network). In contrast, when language comprehension is accompanied by additional task demands (e.g., making some judgment about the word/sentence, or answering comprehension questions), such paradigms elicit responses in both the language network and the MD network (see Diachek et al., 2020 *J Neuro* for a meta-analysis, and Fedorenko & Shain, 2022 *Persp Psych Sci* for a review).

R2.6: *Related to this, because there were not consistent measures of comprehension associated with the different language tasks, it is difficult to assess to what extent participants were attending to the stimuli, which is likely to affect activation patterns.*

RESPONSE R2.6:

Thank you for raising this point. You are absolutely right that passive language comprehension paradigms, while addressing the concern of conflating linguistic processing and task-related cognitive demands (discussed just above), has a potential downside of no behavioral measures of comprehension. The lack of such measures would make it difficult to interpret the lack of a response to the Language>Control contrast: i.e., is a participant not showing a response because their language processing mechanisms are immature? because they are not awake or paying attention? because they don't understand the input? However, in the presence of *robust responses to the Language>Control contrast* (which we observe in all age groups in both datasets), this concern is alleviated: there is clearly more neural activity happening during the Language condition compared to the Control condition, and a large body of prior work (e.g., reviewed in Fedorenko et al., 2024, *NRN*) justifies the interpretation of this activity as reflecting linguistic computations (e.g., in adults, we see the same level of response in these language areas for minimally different paradigms: with and without an accompanying task; Fedorenko et al., 2010 *J Neurophys;* Scott et al., 2017 *Cog Neuro*).

That said, these concerns about attention may affect the interpretation of the age-related changes in the magnitude of response to language, as we have now acknowledged (lines 484). In particular, although it has been established that some minimal amount of attention is necessary for eliciting a response in the language areas (Cohen et al., 2021, *Cortex*), differences across individuals or groups of individuals in the level of response to language may reflect i) differences in the degree of engagement of linguistic computations, ii) differences in the amount of attention paid during the critical language task, or iii) some combination of the two. One reason why we think the first factor is more likely is that increases in response to language—similar to those that we observe here across development—have been reported in adult second language learners (with fully mature attention mechanisms) as a function of their increasing proficiency in the target language (Malik-Moraleda, Jouravlev et al., 2024 *Cer Cort*), which suggests that the ability to engage the relevant linguistic computations is a more parsimonious account of changes in language response magnitude. We have now made this point briefly in the Discussion (lines 479-487).

R2.7: *The authors also mention the specialization of the right hemisphere for non-linguistic information however they should also consider that the right hemisphere has been shown to process other components of spoken language, such as voice recognition and some types of prosody. (The work of Hickok and Poeppel is highly relevant to this discussion and should be cited).*

RESPONSE R2.7:

Thank you for this comment. We have now added some references to the body of work on the right-hemisphere's contributions to language processing (lines 430-436). (We do point out that because most past work has relied on group analyses, it is difficult to unambiguously attribute those reported effects to the homotopic language areas, as opposed to one of the other large-scale networks interdigitated with the language network on the right-hemisphere lateral frontal and temporal cortical surface.)

R2.8: *In addition, the discussion of continued language development across the age range included in the study is somewhat sparse**.** There are specific language skills that continue to develop at different ages. While speech perception and basic syntactic skills are well in place by preschool age, higher level language skills such as inferencing continue to develop, as well as dramatic increases in vocabulary.*

RESPONSE R2.8:

Thank you. We have now added some references to papers that discuss these different developmental trajectories of different aspects of language, but space constraints do not allow for a more extended discussion (lines 459-460).

Methods:

R2.9*: While I think it is reasonable to combine the different language stimuli and control conditions as the authors have done, it is important for them to clarify that the contrasts are *not* the same across the datasets. Importantly, backward speech contains limited recognizable phonemic information. Degraded speech, as described in the methods, is also unlikely to include recognizable phonemic information this means that when comparing these conditions to normal speech, recognition of speech sounds is a part of the activation in that comparison. In contrast, when using a foreign language as the control condition it is likely that there is overlap in phonemic information in the native language in foreign language.*

RESPONSE R2.9:

Thank you for the chance to clarify this point. You are absolutely right that the *speech processing* demands vary somewhat between some of these control conditions. However, with respect to *language comprehension* (and the brain regions that we focus on), these differences should not play a large role (for example, see Malik-Moraleda, Ayyash et al., 2022, *Nat Neuro* for evidence that individual activation maps for the Language>Degraded contrast and the

Language>Foreign contrast are highly similar). We also believe that the similarity in the key patterns between the two datasets (which use different controls) is a strength, suggesting that these patterns are robust to these differences in the contrast used. All this said, we have acknowledged this issue in the Limitations section (lines 495-497).

R2.10: *The task in dataset two included a probe condition, but performance data is not included and it is unclear whether this varied with age.*

RESPONSE R2.10:

Thank you. We have now included the behavioral data analyses in **SI-1B** (SI lines 129-148) and referenced it in the main text (line 590). In this task, participants listened to a 20-second story clips, and then were asked whether a 3 second probe sentence would come next in the story indicating "Yes" or "No" with a button press. As expected, response accuracies increased with age. Statistically, the adult group performed significantly better than the early group (p = 0.05) and the middle group (p < 0.05). No other differences were reliable (see **Table SI-1B;** SI line 144).

R2.11: *While the analysis approach is generally well motivated, I am concerned about the effect of choosing the top 10% of activated voxels within each parcel as individually-defined functional ROIs. This could be clarified with some examples of how this would look on an individual level and some quantitative data on numbers of voxels involved (were they always contiguous within the parcel?). Are the sample activation maps included in Figure 2 thresholded based on this top 10% criteria? It seems like excluding a large number of voxels in each parcel would have a dramatic effect on lateralization calculations, and restricting the analysis to the most active voxels in the parcels in both hemispheres might bias the results. The overlap maps shown in Figure SI-6 do suggest that areas of activation in children largely overlapped with those of adults, but it is unclear how spatially consistent this was at the individual level, and how this would affect lateralization calculations. Much clarification is needed on this aspect of the methods.*

RESPONSE R2.11:

Thank you for this comment. In selecting the top 10% of activated voxels, we are simply following the approach we have been using with adults over the last decade (where we have established that top 10% correspond closely to selecting voxels at a fixed significance threshold of p<0.001; e.g., Lipkin et al., 2022). Following the standard approaches in visual neuroscience, where the functional localization approach is the gold standard, we do not impose spatial contiguity constraints on the fROIs; in most cases, in both adults and children, the fROI within each parcel consists of a large contiguous cluster of voxels with a few additional smaller clusters.

We have now provided a new SI figure showing sample fROIs across age groups (**SI-2C;** SI line 201; Referenced in main manuscript on line 740). We also would like to point out that whereas our magnitude-based measure of lateralization uses the top 10% fROIs, the complementary, volume-based measure uses a fixed statistical threshold, and the newly-added LI measure (developed by Wilke; **SI-3C;** SI line 360; Brief results in the main manuscript: lines 260-264;

Brief methods in the main manuscript: lines 777-779; 825-827) uses adaptive thresholds. The fact that the results converge across these different measures suggests that the details of how the fROIs are defined does not affect the key findings (in line with extensive explorations of these choices in prior work on adults).

R2.12: *It is briefly noted that not all participants were native speakers of English and some participants were tested in other languages. In some ways this is a strength of the study because the results are not limited to English, however this language diversity was included only for adult participants and not for children, so it represents a confound when looking at potential changes across age. In addition, the degree of bilingualism of participants is not described.*

RESPONSE R2.12:

Thank you for this question. Indeed, the adult group in Dataset 1 comes from a study where we tested native speakers of diverse languages (Malik-Moraleda, Ayyash et al., 2022, *Nat Neuro*). However, it is not clear to us how this difference could be a "confound" (i.e., provide an alternative interpretation for the observed differences). In particular, the adult group serves as a control population in the current study; the magnitude of response to different languages appears to be similar when the materials are similar in complexity and only depends on how proficient one is in the language (e.g., Malik-Moraleda, Jouravlev et al., 2022 *Cer Cort*) (here, we are looking at the responses to one's *native language* in all cases). Furthermore, the magnitude of response to one's native language is highly similar between *monolinguals and bilinguals*: for example, we are pasting below a figure from Malik-Moraleda et al. (2021, *Neurobio Lang),* which illustrates this point for relatively large (n=~50 in each group) and closely matched groups of bilingual and monolingual participants (the blue bars are responses to a non-linguistic task, so are not of interest here). We also have data from another smaller group of adults who have performed an auditory version of the English language localizer, and the magnitude is near-identical to the one in the currently used larger group; because we think larger samples are generally better and the results don't seem to be affected by the language (diverse languages vs. English) or bilingualism of the participants, we chose to keep using the larger sample of adults for DS1.

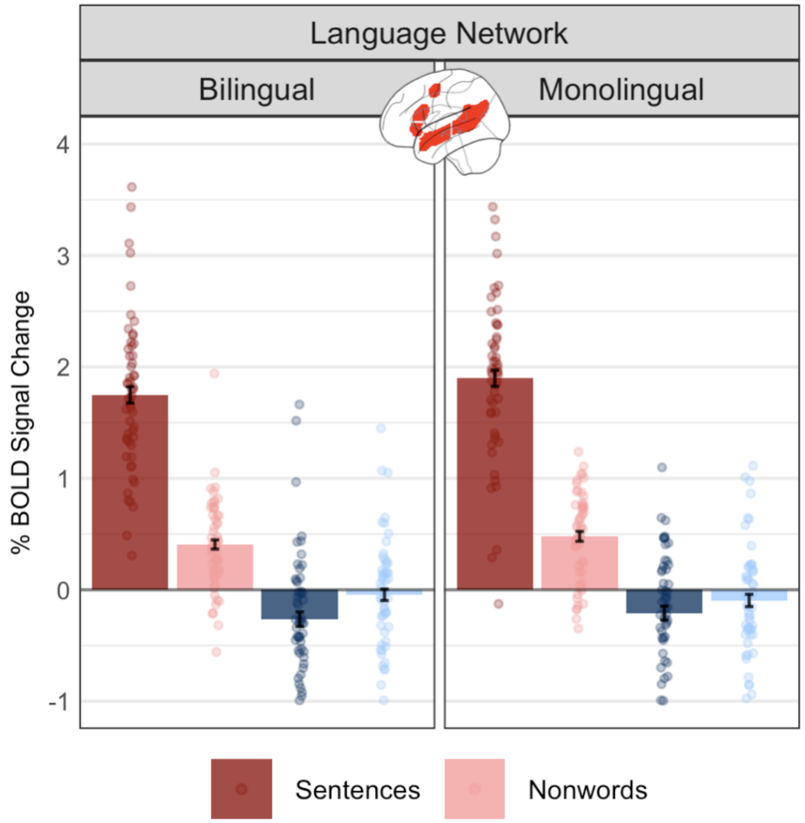

Reviewer #3:

Overview:
This paper reports on an investigation of language localization in predominantly English-speaking individuals aged between 4 and 60 years. Localization was based on three passive (i.e., listening) and one more active (comprehension: Yes-No decision to consistency of story segments) tasks. Overall results suggest similar bias across multiple age groups.

Dear Dr Ozernov-Palchik and colleagues,
Thank you for the complex and thorough work. It's great to see people approaching this question from multiple angles. The work appears to be carefully conducted, and the paper is clearly written, well presented, and will be a great contribution to the field. I have a few general comments and a series of specific comments that may have a bearing on the presentation of the work. I hope these are helpful.
Best wishes,

Thank you so much for your positive comments and very helpful questions and suggestions. We have addressed/incorporated those in the revision, as summarized below.

R3.1: *Given the language listening and comprehension tasks used, it would be worth considering whether the general claims about the language network should be tempered. The justification of*

the use of these tasks over productive tasks is sound, but given the breadth of the language system (Poeppel, 2003; Poeppel et al., 2012; Poeppel & Hickok, 2004), being specific about the claims feels important.

RESPONSE R3.1:

Thank you for the chance to clarify. This point echoes some comments from Reviewer 2.

We assume that by "productive tasks" you mean tasks that involve language production? The system that we focus on (what we refer to as the "language network") supports both comprehension and production as has now been established across many studies (e.g., Menenti et al., 2010, *Psych Sci*; Silbert et al., 2014, *PNAS*; Hu, Small et al., 2022, *Cer Cort*; Zada et al., 2024, *Neuron*; see Fedorenko, Ivanova, & Regev, 2024 *Nat Rev Neurosci* for a review). Furthermore, much work on this network has been carried out over the last ~15 years, and the papers you refer to do not yet incorporate this body of work that has emerged more recently. All this said, you are of course right that the developmental trajectory of the neural infrastructure for language production may well be different, even though the same underlying brain regions are implicated. We have now acknowledged this point in the Limitations section (lines 495-497).

***R3.2**: In addition to the above comment, having multiple tasks to interrogate for the language network would be a helpful consideration for future work (Parker et al., 2022). There may be some covert production tasks that could be used in future research (Bookheimer et al., 1998; Wilke et al., 2005, 2010). Consider including reference to these in the current discussion.*

RESPONSE R3.2:

Thank you. We agree that having multiple language tasks tapping different aspects of language processing would be hugely helpful – we have added this point to the Limitations section (≈).

***R3.3**: It would be useful to know whether the methods developed by Wilke and Lidzba (2007) were considered for the LI calculation. It feels like a robust technique that would be appropriate for this work.*

RESPONSE R3.3:

Thank you for bringing up the approach developed by Wilke and Lidzba (2007). Their method has several strengths, including the use of adaptive thresholding based on average voxel intensities, which accounts for variability across datasets and minimizes the influence of noise. We have now performed a new analysis using the LI toolbox in SPM (developed and maintained by Wilke) to complement our primary analysis (lines 774-779). We utilized the recommended best practice methods with this toolbox, including the use of the standard bootstrapping and adaptive thresholding approach (10,000 indices calculated at each threshold) for the "voxel values" (i.e., response magnitude). We applied our standard bilateral language parcels to mask the data (i.e., inclusive mask), and did not apply any additional masking (e.g., exclusive masking). Statistics were calculated with each individuals' overall lateralization index.

Our primary finding–that the lateralization of the childhood groups does not differ significantly from the adult group–remains robustly present in both datasets with the use of this toolbox. An ANOVA model predicting lateralization index from age, with adults as the reference age group, reveals that none of the childhood groups differ significantly from the adult group in either dataset. We have added these figures and results to Supplementary Information (**SI-3C;** SI line 360). (Brief results in the main manuscript: lines 260-264; Brief methods in the main manuscript: lines 777-779; 825-827.)

We think that this additional analysis strengthens the paper, so thank you very much for suggesting it.

R3.4. *Lateralisation development and neuropsychology*
The introduction states that bilateral language activation during early childhood has been proposed because early left-hemispheric brain damage can leave language relatively unimpaired. However, all but one of the studies cited (references 13-18) investigate children with damage acquired before or within one year of birth. Since this studies' sample are 4+ years old, its findings don't seem to contradict the possible bilateral language representation as much as the discussion suggests it does (line 434). The samples' being too old is mentioned briefly in the discussion, but I would suggest either including age of early left-hemisphere damage being generally below four as an explanation for the discrepancy, or highlighting examples where the damage occurred closer to the age of 4.

RESPONSE R3.4:

Thank you for highlighting the importance of considering the age at which left-hemisphere damage occurs. You are right that the studies we originally cited have indeed focused on injuries at or around birth, but some studies have found that children who sustained left-hemisphere injury at an older age (including beyond age 4 years) can still exhibit a compensatory shift toward the right-hemisphere language areas (e.g., Lidzba, Kupper et al., 2017; Garriz-Luis et al., 2021). We have revised the text to more clearly and accurately discuss this body of relevant prior work (lines 65-67; 355-359). We do emphasize that past brain imaging evidence that has been used to argue for more bilateral language processing in children (e.g., Berl et al., 2012; Holland et al., 2007; Olulade et al., 2020) has only examined children aged 4 and older (line 73), and we highlight the possibility of more bilateral responses at a younger age than what has typically been examined in developmental studies of language processing (lines 424-426).

R3.5: *Methods for estimating lateralisation*
Three methods of estimating lateralisation based on the fMRI data are included, but it would be good to see some rationale/explanation for why all three methods were used, and maybe some assessment of the merits of each for assessing lateralisation. Something to consider is that voxel counting might lead to an overestimation of the degree of lateralisation (Bradshaw et al., 2017; Jansen et al., 2006).

RESPONSE R3.5:

Thank you for this comment. Each measure has its advantages but also limitations. As you noted, voxel-count-based measures can inflate lateralization or otherwise lead to variable results depending on the threshold (see Wilke & Lidzba, 2007 for discussion), whereas magnitude-based measures can be sensitive to outliers. In general, a robust result should come out consistently across multiple measures. As a result, as recommended by recent consensus guidelines on best practices in laterality research (Vingerhoets et al., 2023), we chose to examine and report both volume- and magnitude-based measures, which converge on the same answer: no age-related increase in lateralization. (In the original manuscript, we had also included a measure based on functional connectivity; however, because we only had those data for one dataset and because the set of analyses included in this manuscript is quite extensive, we removed those analyses from the revised manuscript, although the data are available on OSF, and other researchers can perform additional analyses if they wish.)

Specific comments:
R3.6*: Page 21, line 596: "After excluding 31 child participants and 0 adult participants" > the exclusion of 0 adults is mentioned here for Dataset 2. For consistency, it would be best to mention with for Dataset 1 as well (line 586)*

RESPONSE R3.6: Thank you, fixed (line 555).

R3.7*: Page 27, lines 861-862: "For magnitude values, there were two subjects with negative values (one in DS1 and one in DS2)." > This sounds like only 2 subjects were right lateralized for language processing. We would expect more than this in a random sample. Please check to ensure that this is accurate. Alternatively, please clarify in text if I have misinterpreted this value.*

RESPONSE R3.7: Your interpretation is correct, and we have double checked to confirm this is accurate for our dataset. Of note, we are no longer using the LI method for effect magnitude in the main text.

R3.8: *Page 27, lines 862-864: "To allow for LI calculation, we transformed the mean values for each hemisphere by adding the absolute value of the lowest negative number across all fROIs to all values separately for DS1 and DS2." > Can you clarify in text why this transformation was necessary?*

RESPONSE R3.8: Thank you for the chance to clarify. Following R1's recommendation, we no longer rely on this approach, and instead use a regression model that includes hemisphere and age, and their interaction, as predictors of the Language>Control effect size; this approach is conceptually simpler and more robust.

R3.9: *Page 12, Figure 3 > It would be helpful to match the y-axes for the top two panels, magnitude for Datasets 1 and 2.*

RESPONSE R3.9: Thank you. We have revised this figure and have ensured that the axes are consistent (line 278).

R3.10: *Page 12, line 296: "we used the magnitude of the top 10% of tvalues from the Language>Control" > hyphen missing in 't-values' here: just flagging, could be a personal preference or journal requirement. Please check.*

RESPONSE R3.10: Thank you, we've changed the wording on this page, but have ensured that all mentions of 't-values' are hyphenated.

R3.11: *Page 22, line 556: "developmental brain disorders104,105 (see106 for a meta-analysis)" > given that these papers exclusively refer to autism spectrum disorder, the sentence might as well specify this group.*

RESPONSE 3.11: Thank you; we have specified the population, and added a few references to other conditions (line 446)

R3.12: *Page 3, lines 91-92: The sample is described as a 'pediatric cohort' throughout the text, but both datasets include child and adult participants, so that is not an appropriate label.*

RESPONSE 3.12: Thank you, we have removed the word "pediatric" from when referring to the full datasets, and instead refer to these childhood groups as "developmental" or "child" groups to avoid confusion (e.g., line 95).

R3.13: *Page 21, 578-584: It is also not clear if the participants were recruited from a patient population, which 'pediatric' seems to imply. There should be more details about how each dataset was recruited.*

RESPONSE 3.13: Participants were not recruited from a patient population. We have added additional details about participant recruitment in Methods (lines 539-542), and have removed the use of "pediatric" in the manuscript to avoid confusion.

References

Albers, C., & Lakens, D. (2018). When power analyses based on pilot data are biased: Inaccurate effect size estimators and follow-up bias. Journal of Experimental Social Psychology, 74, 187-195.

Bookheimer, S. Y., Zeffiro, T. A., Blaxton, T. A., Gaillard, W. D., Malow, B., & Theodore, W. H. (1998). Regional cerebral blood flow during auditory responsive naming: Evidence for cross-modality neural activation. Neuroreport, 9(10), 2409–2413.

Bradshaw, A. R., Bishop, D. V. M., & Woodhead, Z. V. J. (2017). Methodological considerations in assessment of language lateralisation with fMRI: A systematic review. PeerJ, 5, e3557. https://doi.org/10.7717/peerj.3557

Dienes, Z. (2014). Using Bayes to get the most out of non-significant results. Frontiers in psychology, 5, 781.

Hoenig, J. M., & Heisey, D. M. (2001). The abuse of power: the pervasive fallacy of power calculations for data analysis. The American Statistician, 55(1), 19-24.

Jansen, A., Menke, R., Sommer, J., Förster, A. F., Bruchmann, S., Hempleman, J., Weber, B., & Knecht, S. (2006). The assessment of hemispheric lateralization in functional MRI—Robustness and reproducibility. NeuroImage, 33(1), 204–217. https://doi.org/10.1016/j.neuroimage.2006.06.019

Lakens, D. (2022). Sample size justification. Collabra: Psychology, 8(1), 33267.

Levine, M., & Ensom, M. H. (2001). Post hoc power analysis: an idea whose time has passed? Pharmacotherapy, 21(4), 405-409.

O'Keefe, D. J. (2007). Post hoc power, observed power, a priori power, retrospective power, prospective power, achieved power: sorting out appropriate uses of statistical power analyses. Communication Methods and Measures, 1(4), 291-299.

Parker, A. J., Woodhead, Z. V. J., Carey, D. P., Groen, M. A., Gutierrez-Sigut, E., Hodgson, J., Hudson, J., Karlsson, E. M., MacSweeney, M., Payne, H., Simpson, N., Thompson, P. A., Watkins, K. E., Egan, C., Grant, J. H., Harte, S., Hudson, B. T., Sablik, M., Badcock, N. A., & Bishop, D. V. M. (2022). Inconsistent language lateralisation—Testing the dissociable language laterality hypothesis using behaviour and lateralised cerebral blood flow. Cortex. https://doi.org/10.1016/j.cortex.2022.05.013

Poeppel, D. (2003). The analysis of speech in different temporal integration windows: Cerebral lateralization as ‘asymmetric sampling in time’. Speech Communication, 41(1), 245–255. https://doi.org/10.1016/S0167-6393(02)00107-3

Poeppel, D., & Hickok, G. (2004). Towards a new functional anatomy of language. Cognition, 92(1–2), 1–12. https://doi.org/10.1016/j.cognition.2003.11.001

Poeppel, D., Emmorey, K., Hickok, G., & Pylkkänen, L. (2012). Towards a New Neurobiology of Language. The Journal of Neuroscience, 32(41), 14125–14131. https://doi.org/10.1523/JNEUROSCI.3244-12.2012

Senn, S. (2002). Power is indeed irrelevant in interpreting completed studies. BMJ, 325(7375), 1304.

Vingerhoets, G., Verhelst, H., Gerrits, R., Badcock, N., Bishop, D. V., Carey, D., ... & LICI consortium. (2023). Laterality indices consensus initiative (LICI): A Delphi expert survey report on recommendations to record, assess, and report asymmetry in human behavioural and brain research. Laterality, 28(2-3), 122-191.

Wagenmakers, E. J. (2007). A practical solution to the pervasive problems of p values. Psychonomic bulletin & review, 14(5), 779-804.
Wilke, M., & Lidzba, K. (2007). LI-tool: A new toolbox to assess lateralization in functional MR-data. Journal of Neuroscience Methods, 163(1), 128–136. https://doi.org/10.1016/j.jneumeth.2007.01.026
Wilke, M., Lidzba, K., Staudt, M., Buchenau, K., Grodd, W., & Krägeloh-mann, I. (2005). Comprehensive language mapping in children, using functional magnetic resonance imaging: What's missing counts. Neuroreport, 16(9), 915–919.
Wilke, M., Pieper, T., Lindner, K., Dushe, T., Holthausen, H., & Krägeloh-Mann, I. (2010). Why one task is not enough: Functional MRI for atypical language organization in two children. European Journal of Paediatric Neurology, 14(6), 474–478. https://doi.org/10.1016/j.ejpn.2010.05.002
Zhang, Y., Hedo, R., Rivera, A., Rull, R., Richardson, S., & Tu, X. M. (2019). Post hoc power analysis: is it an informative and meaningful analysis? General Psychiatry, 32(4), e100069.

Response to Reviewers

Reviewer 1

The authors have been highly responsive to suggestions for revision and incorporated additional analyses in their revision. I have also looked at the deposited code and data which allow one to reconstruct the analyses. That is greatly appreciated. Just to add, re statistical power - I was not recommending a post hoc power analysis in my original report, but rather using simulation to obtain power estimates. Apologies if that was unclear. Although that was not done but I am happy to go ahead and recommend publication: this study clearly advances our understanding of this topic.

I have not worked through all the code but have satisfied myself that the results are replicable using the deposited data and code.

Author Response:

Thank you for taking the time to provide such comprehensive and thoughtful feedback. We believe that this has considerably improved the quality and impact of the manuscript.

Reviewer 2

Overall, the authors have been highly responsive to the reviews and I am enthusiastic about seeing this work move forward for publication. I had one minor point remaining:

*- The revision clarified my understanding about selection of the top 10% of voxels in each ROI, however, I still feel the explanation in the methods section is confusing. On page 23 of the main document, the authors state "Individual fROIs were defined by selecting—within each parcel—the 10% of most localizer-responsive voxels based on the t-values for the Language>Control contrast". To me this implied that only *10% of the voxels in each parcel* were selected, which led to my original concern about this method. In the supplementary information, the authors explain that they "selected the top 10% of voxels across the brain for the Language > Control contrast" and then divided up the selected regions according to each parcel. I think the explanation in the supplementary information is clearer and should be carried over to the main manuscript.*

Author Response

Thank you for this thoughtful and constructive comment and for the opportunity to clarify this point. We believe the concern arises from a conflation of two distinct steps in our analysis pipeline, which we clarify below.

First, during parcel derivation and validation using the Group-Constrained Subject-Specific (GSS) analysis reported in SI-2A, we selected the top 10% of voxels across the brain for the *Language > Control* contrast as part of the watershed-based parcellation procedure. This step was used solely to generate developmental parcellations and to compare them with previously established adult parcellations.

Second, for the definition of individual functional ROIs used in all primary analyses, we followed the standard GSS procedure by selecting, within each parcel, the top 10% most localizer-responsive voxels based on the *Language > Control* t-values. This approach defines participant-specific fROIs in both hemispheres, with right-hemisphere fROIs determined using homologous projected parcels.

Thus, the statement that we selected the top 10% of voxels across the brain and subsequently divided those regions by parcel applies to the parcel-derivation validation analysis in SI-2A, rather than to the fROI definition used in the main analyses.

To prevent potential confusion for readers, we have revised the text in SI-2A to state explicitly: *"Importantly, the across-brain top 10% step is used only for parcel derivation in the GSS validation analysis. All primary fROI definitions in the main analyses select the top 10% of voxels within each parcel."*

We hope this clarification resolves the concern, and we thank the reviewer for prompting us to make this distinction clearer in the manuscript.
